# GRU-SCANET: unleashing the power of GRU-based sinusoidal capture network for precision-driven named entity recognition

**DOI:** 10.1093/bioadv/vbaf096

**Published:** 2025-06-16

**Authors:** Bill Gates Happi Happi, Geraud Fokou Pelap, Danai Symeonidou, Pierre Larmande

**Affiliations:** DIADE, IRD, CIRAD, University of Montpellier, Montpellier 34394, France; LIRMM, CNRS, INRIA, University of Montpellier, Montpellier 34095, France; URIFIA, Department of Computer Science, University of Dschang, Dschang 00237, Cameroon; MISTEA, INRAE, 2 place pierre viala, Montpellier 34060, France; DIADE, IRD, CIRAD, University of Montpellier, Montpellier 34394, France; LIRMM, CNRS, INRIA, University of Montpellier, Montpellier 34095, France

## Abstract

**Motivation:**

Pre-trained Language Models (PLMs) have achieved remarkable performance across various natural language processing tasks. However, they encounter challenges in biomedical named entity recognition (NER), such as high computational costs and the need for complex fine-tuning. These limitations hinder the efficient recognition of biological entities, especially within specialized corpora. To address these issues, we introduce GRU-SCANET (Gated Recurrent Unit-based Sinusoidal Capture Network), a novel architecture that directly models the relationship between input tokens and entity classes. Our approach offers a computationally efficient alternative for extracting biological entities by capturing contextual dependencies within biomedical texts.

**Results:**

GRU-SCANET combines positional encoding, bidirectional GRUs (BiGRUs), an attention-based encoder, and a conditional random field (CRF) decoder to achieve high precision in entity labeling. This design effectively mitigates the challenges posed by unbalanced data across multiple corpora. Our model consistently outperforms leading benchmarks, achieving better performance than BioBERT (8/8 evaluations), PubMedBERT (5/5 evaluations), and the previous state-of-the-art (SOTA) models (8/8 evaluations), including Bern2 (5/5 evaluations). These results highlight the strength of our approach in capturing token-entity relationships more effectively than existing methods, advancing the state of biomedicalNER.

**Availability and implementation:**

https://github.com/ANR-DIG-AI/GRU-SCANET.

## 1 Introduction

Named entity recognition (NER) is pivotal for many natural language processing (NLP) and knowledge acquisition tasks. Typically, the task of NER is to identify real-world entities in an unstructured text using categories such as person, location, organization, and time. NER is also needed as an initial step in processes requiring question–answering (Liu *et al.* 2022, Shrimal *et al.* 2022), information retrieval (Spositto *et al.* 2022, Zheng *et al.* 2022), co-reference resolution (Strobl *et al.* 2022), topic modeling (Aguiar *et al.* 2022, Joshi *et al.* 2022), domain expert assistance (Lo *et al.* 2022, Zhang *et al.* 2022b), to name a few. Machine learning and deep learning play an important role in the biological domains to address various challenges (Sapoval *et al.* 2022). Recent advances in NLP, such as transformers (Vaswani *et al.* 2017), have significantly improved NER performance, particularly in biomedical contexts, where many entities share similar naming conventions across different species and disciplines (Shen *et al.* 2017, Catelli *et al.* 2020). Models like Long Short-Term Memory (LSTM) (Kratzert *et al.* 2021) and conditional random field (CRF) (Lafferty *et al.* 2001) have also greatly improved performance in biomedical NER over the last few years (Habibi *et al.* 2017, Giorgi and Bader 2018, Yoon *et al.* 2019, Song *et al.* 2021).

Additionally, word embeddings, such as Word2Vec (Zhang *et al.* 2022a, Mikolov *et al.* 2013), play a crucial role in capturing token semantics by learning from extensive text corpora such as English Wikipedia, PubMed abstracts, and PMC enabling better contextual understanding and token similarity detection. In addition, these embeddings can automatically detect semantic similarities (Kiela *et al.* 2015). For instance, if a model learns the “New York” token in certain contexts and relationships, it can also recognize “NY” as a similarly named entity. However, pre-trained embeddings based on general corpora may reduce the effectiveness of applying NER to domain-specific tasks. This can lead to an information overload for entity recognition within a specific domain, raising concerns about the sensitivity to the quality of embeddings used in this new domain (Günther *et al.* 2020). A recent survey on NER by (Maud Ehrmann *et al.* 2023) exposes the problem of lack or imbalanced resources that can influence the capability of the model to perform an accurate prediction.

Therefore, for some architecture, obtaining high-quality embeddings often requires the utilization of large text corpora. For example, BioBERT (Lee *et al.* 2020) has been trained on large-scale biomedical corpora comprising approximately 4.5 billion tokens from PubMed abstracts and 13.5 billion tokens from PMC full-text articles. While large-scale language models like BioBERT achieve notable results, their training requires substantial computational resources and fine-tuning, and their performance gains over general NER models can be modest (Li *et al.* 2022). Furthermore, the reliance on fixed training data introduces biases, as model predictions are influenced by the unbalanced context distributions within the corpora (Lee *et al.* 2019).

Although Large Language Models (LLMs) like GPT have revolutionized NLP, their performance in predicting the next token is not without limits (Lai *et al.* 2023). Indeed, their token compression techniques, such as Byte Pair Encoding (BPE), may hide crucial contextual information and introduce bias into the prediction (Bostrom and Durrett 2020), particularly in biomedical NER tasks, where precise token-entity relationships are essential. These biases could result from an unbalanced distribution of contexts in the training data. Additionally, LLMs can struggle with capturing the nuanced relationships between input tokens and output tags, which is necessary for achieving high accuracy in NER (Li *et al.* 2024).

This article presents GRU-SCANET (Gated Recurrent Unit-based Sinusoidal Capture Network), a novel architecture designed to efficiently capture the relationships between input tokens and output tags based on the sentence context. Unlike existing models that rely heavily on large-scale pre-training, GRU-SCANET leverages a lightweight structure utilizing positional encoding to capture token positions, a Bidirectional GRU (BiGru) (Abdelgwad *et al.* 2022) for learning contextual representations, an attention-based encoder to capture token relations, and a CRF decoder for accurate entity labeling. Our architecture was evaluated on eight biological NER datasets, demonstrating robust performance across varied corpora and outperforming state-of-the-art models. The results validate the effectiveness of GRU-SCANET in addressing challenges related to unbalanced datasets while maintaining computational efficiency.

The rest of this article is organized as follows. Section 2 presents the problem formulation. Section 3 presents the GRU-SCANET architecture. Section 4 provides implementation details. Section 5 reports evaluation results and discusses performance compared to other models.

## 2 Methods

### 2.1 NER problem and formalization

NER models based on Bidirectional Encoder Representations from Transformers (BERT) or Generative Pre-trained Transformer (GPT) architectures require extremely costly pre-training on corpora and significant processing time for simple tasks to ensure token memorization within various contexts of use (Lee *et al.* 2019). The performance of these models may be degraded if the large corpora on which they rely do not provide a wide enough range or an adequate number of contexts for tokens whose vector representations are critical (Dai *et al.* 2019). For models like BERT and GPT, the absence of context to enrich token representations could make NER processes less effective than expected due to the imbalanced meaningful token representations appearing in the context to predict the entity type of the next token in the sequence. Thus, we believe it would be preferable to learn how to handle the NER task by directly capturing the relationships between the entities to be detected and their appearances within their contexts to predict them effectively.

**Figure 1.**
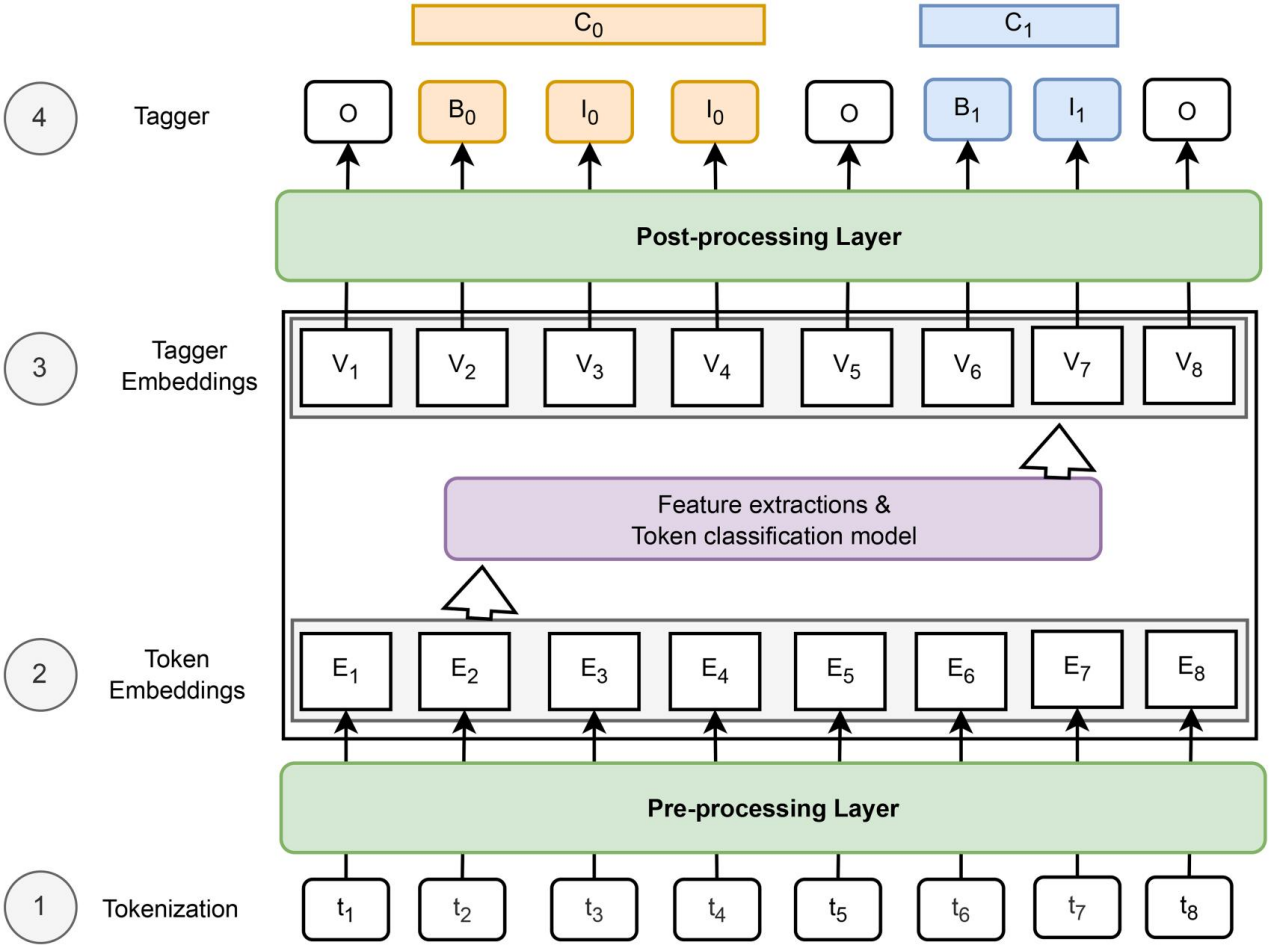
General overview of supervised NER process.

Figure 1 represents the four successive steps leading to performing the NER process. (i) Tokenization: The input sentence is meticulously divided into individual tokens, laying the foundation for further analysis. (ii) Token Embeddings: Each token embarks on a journey into a high-dimensional vector space, where it is transformed into a unique numerical representation, capturing its semantic essence. (iii) Feature Extraction: These token vectors are then skillfully combined to create comprehensive features, meticulously crafted to represent the context of each token within the sentence. (iv) Classification: A sophisticated classification model emerges, armed with the extracted features, ready to assign NER tags to each token, identifying the named entities that reside within the sentence.

**Figure 2.**
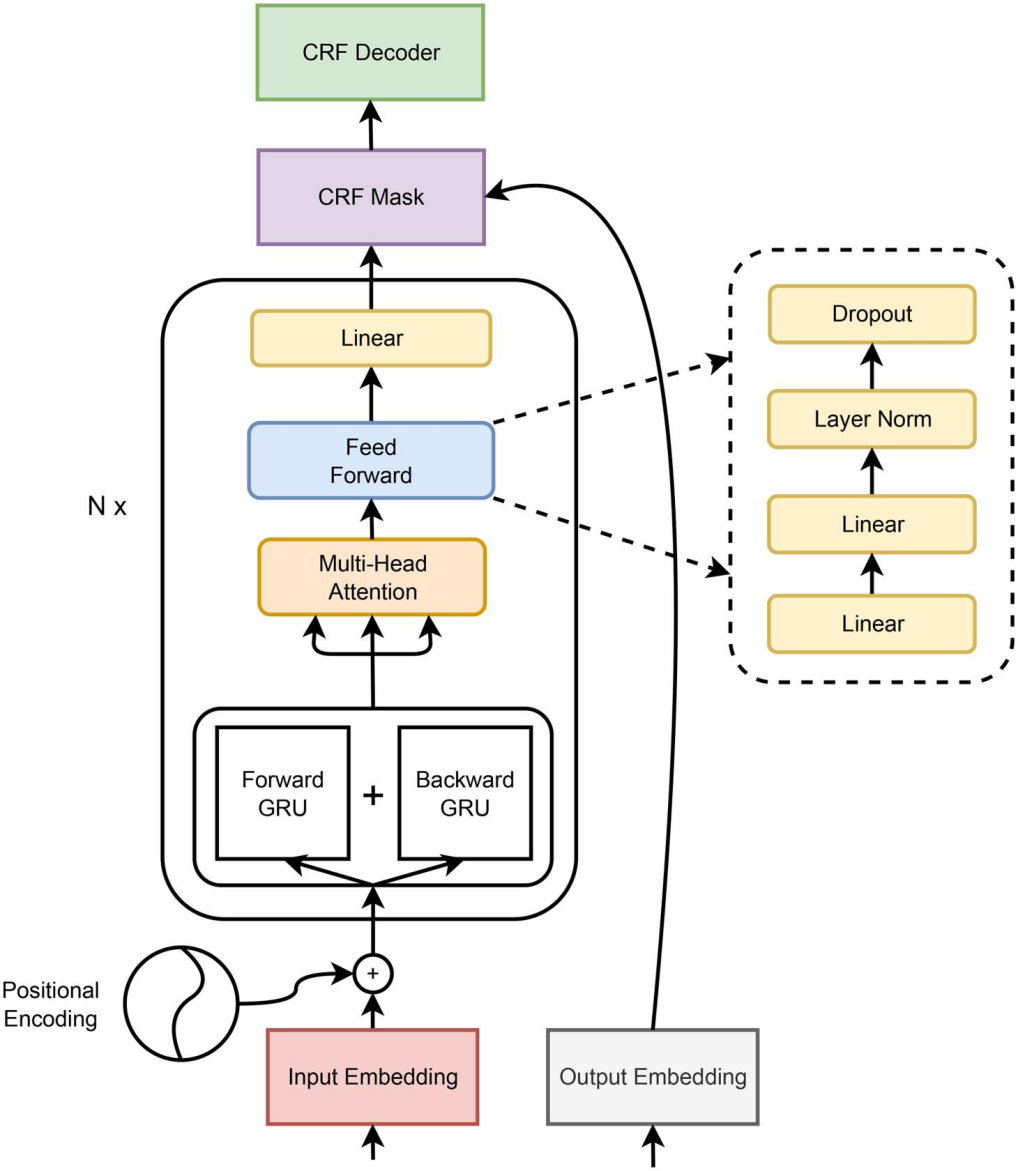
GRU-SCANET architecture.

**Figure 3.**
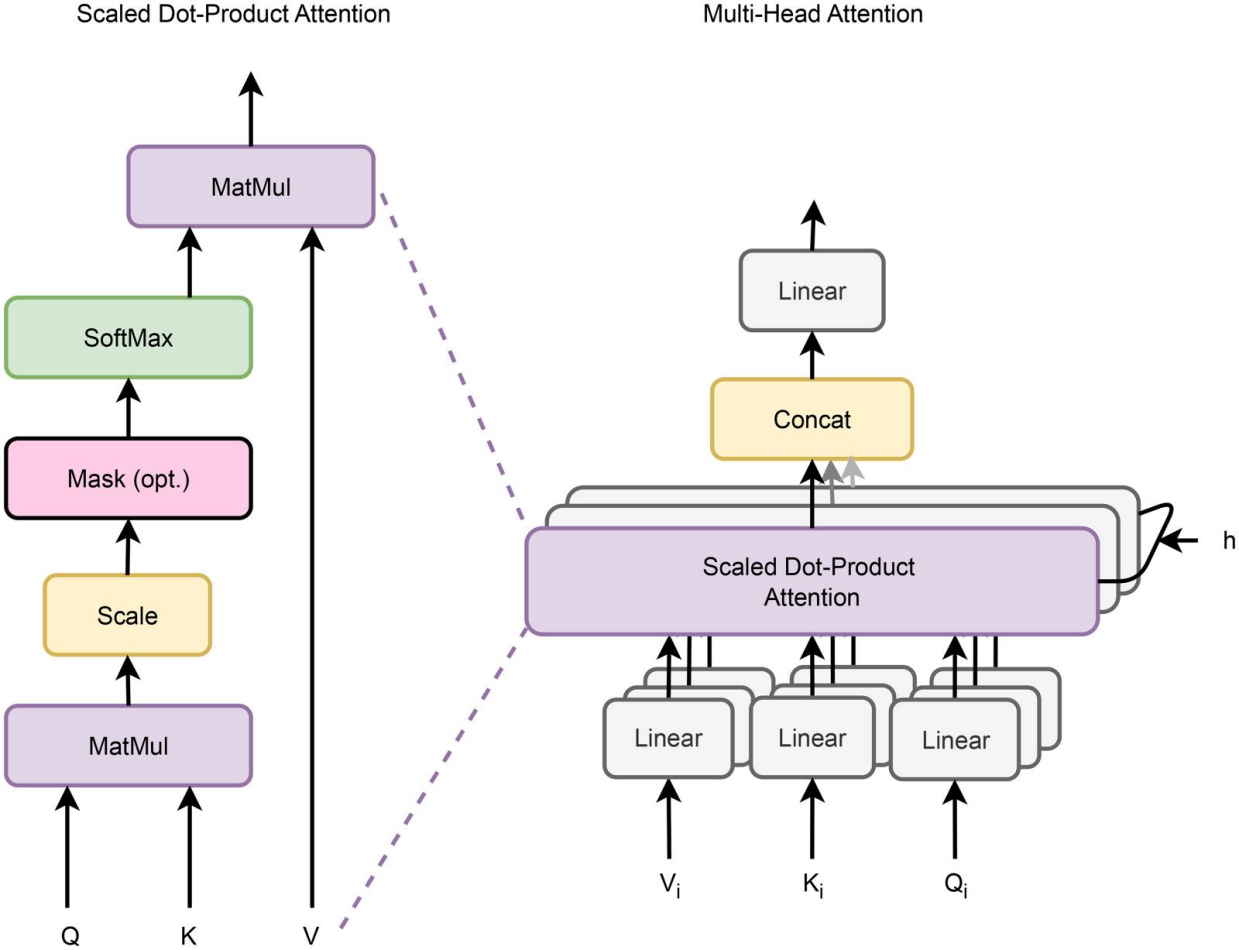
Graphical representation of Multi-Head Attention (Vaswani *et al.* 2017).

Consider an input text, which is a sequence of tokens. Each token in this sequence belongs to a token set (*T*) and is associated with a unique index (embedding or order number). Let *X* be a sequence of tokens represented as $X=[t_{1},t_{2},t_{3},\ldots ,t_{i},\ldots ,t_{n}]$, where *n* is the number of tokens in the sequence ($t_{i}\in T$). Moreover, consider a set of *M* possible entity classes to recognize within *X*. Let us denote as $C_{1},C_{2},\ldots ,C_{M}$ the *M* possible classes to detect, where *M* is the total number of classes. Each entity class, $C_{j}$, is associated with three specific tags:

-
$B_{j}$ (Beginning): This tag is used to mark the beginning of a group of tokens that refer to an entity of class $C_{j}$ in the sequence *X*;
-
$I_{j}$ (Inside): This tag is used to mark a group of tokens in *X* that are part of an entity of class $C_{j}$, except for the first token;
- *O* (Outside): This tag is used to mark a group of tokens in *X* that are not part of an entity class $C_{j}$.

The NER problem involves the building of a model (from an architecture) able to associate each token in *X* with one of the three tags ($B_{j}$, $I_{j}$, O) of each of the *M* entity classes, to identify and annotate entities within *X*. The $V_{i}$ of tags ($B_{j}$, $I_{j}$, O) are identical on the training data and different during the inference phase due to the probability estimations. In the following sections, we will provide a detailed explanation of each component in our model.

### 2.2 Approach

GRU-SCANET (Figure 2) was trained and evaluated on eight different biological datasets that were built from the BioCreative benchmarks (https://github.com/dmis-lab/biobert). We give the main stages of treatment and their essential points in the following:

1. *Inputs:* from the datasets (test and train from each dataset), we produce a padded *d*-dimensional vector representation of each sentence with their index from the set of words as formalized in Section 2 under a form of an integer list. To enhance the high ability of the model to associate word subgroups with their corresponding entity classes, each padded input vector is combined with a positional encoding vector of the same dimension. This addition incorporates the numerical identifier of a word and its position within the input word sequence, effectively specializing the context in which the word is used. This approach significantly improves the distribution of conditional probabilities within the model architecture.
2. *Model Construction:* After forwarding inputs through BiGRU (Abdelgwad *et al.* 2022) operations, we applied transformation layers. These layers associate the input (word index vector and its positional encoding) with its corresponding output, which is represented as a vector of expected numerical delimiter tag in the output for each entity class (as mentioned in Section 2).
3. *Model Training and Testing:* The various datasets are divided into training, development, and test sets. The training step involved optimizing model parameters to minimize prediction errors. There is no need to perform validation steps on development data with GRU-SCANET. Model evaluation was performed on the test set to measure the performance of the model, including precision (P), recall (R), and F1-score (F).
4. *Model Optimization:* By adjusting hyper-parameters such as network size, learning rate, dropout and epochs (Abdelgwad *et al.* 2022), and weights during processing, we improve the model.

First, the training data were created by matching sequences of words with sequences of tags, using pairs of input words and their corresponding output tags. Next, the model was initialized with an encoder (*N* = 1), which captured the contextual information of the input words. The input sequences were encoded using the *N* encoder (which refers to the transformation layer), and then, the CRF decoding layer was initialized with the expected tags. The training process was repeated over multiple epochs, with adjustment of weights with a backpropagation algorithm, until the model achieved satisfactory performance on the test data. In the following section, we go in-depth into the concepts used by GRU-SCANET.

### 2.3 Input embedding and positional encoding

Combining sequence embedding with positional encoding aims to consider the relevance of the tokens in their relative position in the sequence *X*, thereby enhancing the model’s ability to capture contextual and spatial information.

- *Input Embedding:* Consider $T^{*}$ being the set of all the sentences. Each sequence of tokens *X* (*X*$\in T^{*}$) is represented by matrix denoted $E(X)\in R^{d\times m}$ ($d,m\in N^{*})$ in the embedding space. The matrix of *X* is built using the indices of tokens in *T* combined with the function $E_{i}$. The token embedding function defined as $E_{i}:T\to R^{m}$. Each token $t_{i}$ is associated with a *m*-dimensional real-valued vector. E(X) of the input sequences may not always be the same size as *d*. In such cases, padding is necessary. Padding involves adding a special token with a zero *m*-dimensional vector representation. This ensures that all sequences have a uniform length for further processing. For example, considering that $X=[t_{1},\ldots ,t_{8}]$ and d=8 according to Fig. 1, $E(X)=[E_{1}(t_{1}),\ldots ,E_{8}(t_{8})]$ and if d=10, $E(X)=[E_{1}(t_{1}),\ldots ,E_{8}(t_{8}),E_{*}(0),E_{*}(0)]$. Generally, *d* is defined from the size of the longest sequence of input tokens in the annotated training data and *m* is fixed to an integer constant (e.g. m=256).
- *Positional Encoding:* It is typically added to sequence embedding to introduce a notion of position. It uses trigonometric functions to assign specific values to each position in the sequence. A commonly used formula for positional encoding is defined below: Let t(∈N) be the desired position in an input token sentence of size *d*. *d* represents the max length of input token sequences previously seen. $\vec{P_{t}}\in R^{d\times m}$ be the corresponding positional encoding of token in position *t* in *X* (Vaswani *et al.* 2017). *t* belongs to {0,…,d−1}, i(∈N) belongs to {0,…,m−1}, and k∈N.

${\vec{P_{t}}}^{\left(i\right)}=\left\{\begin{array}{cc} \;\text{sin}(w_{k}.t), & if\;i=2k\\ \;\text{cos}(w_{k}.t), & if\;i=2k+1 \end{array}\right\}$ with $w_{k}=\frac{t}{10000^{\frac{i}{d}}}$.

### 2.4 Encoder stack

#### 2.4.1 Forward and backward GRU models

*Note:* the input of this block is the addition member to member of positional encoding and the embedding of *X* ($(\vec{P}+E(X))\in R^{d\times m}$).

The Forward and Backward GRU (Gated Recurrent Unit) models are variations of a type of RNN called BiGRU (Abdelgwad *et al.* 2022). These models process sequence data, such as sentences or temporal sequences. The Forward GRU model is designed to process sequences chronologically, from left to right. It considers the past information to predict future states. At each time step, the Forward GRU receives the current input and the previous hidden state to generate a new output and update its hidden state. On the other hand, the Backward GRU model processes sequences in reverse order, from right to left. It uses future information to predict past states. At each time step, the Backward GRU receives the current input and the next hidden state to generate new output and update its hidden state. Simultaneously using both the Forward and Backward GRU models enables capturing contextual information from the past and future of a sequence. This allows associating the sequence with its corresponding output class, which is then processed by the subsequent layer to ensure chronological attention.

*Note:* the output of this block is $\mathrm{BiGru}(E(X))\in R^{d}$.

#### 2.4.2 Multi-head attention

*Note:* the output of the BiGRU layer is assigned to Q=K=V=BiGru(E(X)).

In the NER context, the multi-head attention mechanism (Figure 3) helps to capture relationships and dependencies between tokens in a sequence to represent named entities. The main parameters of the Multi-Head Attention (MHA) process in transformers architectures include the attention heads (default: *h* = 4) having the respective dimensions $d_{q},d_{k},d_{v}\in N(d_{q}=d_{k}=d_{v}=d/h=256/4=64,$ according to Section 4.1, d=256) for learnable matrices $W_{i}^{q}\in R^{d_{q}\times d}$, $W_{i}^{k}\in R^{d_{k}\times d}$, $W_{i}^{v}\in R^{d_{v}\times d}$. This allows each head to attend to different parts of the input token sequence (from the previous layer) and focus on the non-similar relationships. For each head, the attention scores are calculated by computing the dot product between the query and key representations. The attention scores are then scaled and passed through a softmax function to obtain attention weights. The attention weights compute a weighted sum of the token value representations. This emphasizes the importance of different positions in the input token sequence based on their relevance to the query. The weighted sum is then concatenated across all the heads and projected back to the original dimension using a weight matrix ($W^{o}\in R^{h\times d}$).

Finally, the output of the MHA process is obtained by applying layer normalization and residual connections to the concatenated and projected representation. This helps stabilize the learning process and allows the model to capture the dependencies and relationships within the input sequence.


$$\mathrm{MHA}(Q,K,V)=[\mathrm{hea}d_{1},\mathrm{hea}d_{2},\ldots ,\mathrm{hea}d_{h}]\times W^{o}$$

with $\mathrm{hea}d_{i}=\mathrm{Attention}(Q\times W_{i}^{Q},K\times W_{i}^{K},V\times W_{i}^{V})$

In practical implementation, the attention function is computed on a set of queries packed together into a matrix *Q* (Vaswani *et al.* 2017). The keys and values are also packed together into matrices *K* and *V*. The output matrix is then computed by applying the attention mechanism:


$$\mathrm{Attention}(Q,K,V)=\mathrm{Softmax}(\frac{QK^{T}}{\sqrt{d}})\times V.$$

#### 2.4.3 Feedforward network layer and last linear transformation

The feedforward sub-block, a crucial component of the Transformer architecture, comprises four sequential linear transformation operations. It processes the output from the BiGRU layer and generates a *d*-dimensional vector for the final linear layer of the architecture. The first linear layer within this block employs the ReLU (Agarap 2018) activation function. The hidden dimension for all layers within the feedforward sub-block is set to dff = 512. The feedforward sub-block consists of four linear transformation operations, each represented by a matrix multiplication and a bias addition.


$$\begin{array}{l} x=MHA(Q,K,V)(or\;BiGru(E(X))\;\;\left(\text{after frozen MHA}\right)\\ h_{1}(x)=max(0,x\times W_{1}+b_{1});\\ h_{2}(x)=W_{2}\times h_{1}(x)+b_{2};\\ \tilde{h_{2}}(x)=dropout(h_{2}(x),dropout\_rate);\\ FF_{N}(x)=LayerNorm(\tilde{h_{2}}(x)+x). \end{array}$$

Where $h_{1},h_{2}$ represents the successive linear layers in the sub-block. $W_{1}$ and $W_{2}$$\in R^{h\times d}$. The dropout operation aims to prevent overfitting in neural networks by “dropping out” a certain proportion (dropout rate) of neurons (and their connections) from the network.

Following the feedforward sub-block, a final linearization step is performed using the operation:


$$y=h_{3}(FF_{N}(x))$$

Here, $h_{3}$ represents the final linear layer, $FF_{N}$(*x*) denotes the output of the feedforward sub-block, and *x* represents the input to the sub-block.

### 2.5 Decoder stack: CRF

The CRF decoder’s (Al-Smadi *et al.* 2019) objective is to select the most probable index tag for all the input tokens, considering the input feature scores and index tag transitions. It considers the potential scores of tag sequences generated by the model and the transitions between consecutive index tags. It computes the probability of each index tag sequence using the Viterbi decoding algorithm (Forney 1973).

- *Output Embedding:* Let $T_{g}=\{B_{j};I_{j};O;1\leq j\leq M\}$ be the ordered set of all the taggers of tokens for the *M* classes to detect. Each token $t_{i}$ in the sequence *X* is associated with a numeric-valued tag $o_{i}(o_{i}\in T_{g})$ and generates a *d*-dimensional vector $O_{X}$, which will be utilized by the decoder. $O_{X}$ has same dimension *d* than E(X).
- *CRF Mask:* The CRF mask is applied by multiplying the output vector sequence $O_{X}$ with the binary mask. The masked elements are ignored during the loss calculation and do not contribute to the final predictions. This ensures that only the valid tokens of the sequence are considered during training and prediction, thereby improving the performance and efficiency of the model.

The model is trained in a supervised way by maximizing the probability of correct output tags. This involves jointly optimizing the model weights from the output *y* and the CRF decoder.

The CRF decoder in the architecture allows for the consideration of dependencies between tags, which can improve the coherence of predictions and the overall quality of the model.

## 3 Results

In this section, we aim to demonstrate GRU-SCANET’s ability to maintain its effectiveness with consistent performance when scaled up in the NER recognition process. In Section 5.1, we describe the preprocessing of the datasets. Then, in Section 5.2, we describe the execution environment. In Section 5.3, we present our observations on the performance of GRU-SCANET. In Section 5.4, we present an evaluation of the architecture performance scalability in relation to data size and, consequently, model size. Finally, in Section 5.5, we discuss the key differentiations from existing approaches.

### 3.1 Datasets

We applied a preprocessing step to ensure that the input–output pairs follow the standard problem formulation (see Fig. 1) commonly used in state-of-the-art approaches. This alignment facilitates the model’s learning and prediction during both the training and testing phases and ensures a fair comparison with existing methods. This technique involves formatting the datasets as pairs of text and labels corresponding to individual words or subword sequences.

The datasets used include NCBI Disease (Doğan *et al.* 2014), BC5CDR Disease and Drug/Chem (Li *et al.* 2016), BC4CHEMD (Krallinger *et al.* 2015), BC2GM (Smith *et al.* 2008), JNLPBA (Collier *et al.* 2004), LINNAEUS (Gerner *et al.* 2010), and Species-800 (Pafilis *et al.* 2013). Unlike state-of-the-art models like BioBERT (Lee *et al.* 2019), which rely on bidirectional encoding, or GPT (Lai *et al.* 2023), which uses an auto-regressive process, our architecture does not perform pre-training on diverse corpora to capture token semantics. Instead, it directly maps tokens to the appropriate classes without relying on such extensive pre-training.

Our objective is not to predict masked tokens optimally or adapt them to sequences, but rather to capture the relationships between input and output sequences focused on NER datasets. With GRU-SCANET, there is no need to fine-tune the context for each token with new data, preventing the inclusion of irrelevant information that might affect performance. Our model remains flexible when gradually updated with new data, minimizing disruptions compared to models based on token embeddings, which can be sensitive to statistically underrepresented tokens. During the preprocessing stage, we reorganized the datasets into tokenized sentences paired with their corresponding NER tags, simplifying the subsequent training process.

Moreover, we merged all eight data sources into a single dataset to create a Large Language Model (LLM). Subsequently, each of the eight individual datasets was evaluated by using the generated LLMs. This approach ensures that the models are trained and tested globally on the provided high-quality data without focusing on the specified tasks. More details will be described in Section 5.4.

### 3.2 Experimental setup

The experimental setup for the NER process is based on an architecture comprising one encoder and one decoder. The model is trained using a supervised approach, optimizing the parameters with the Adam optimizer to minimize the loss between the predicted and the ground-truth tags. The environment contains a GPU partition with 468 GB of RAM and eight high-performance GPUs (Nvidia v100). Each compute node is equipped with 64 CPU cores, accompanied by dedicated memory of 7.3 GB per core. To achieve optimal convergence, our training process requires only two iterations. The entire training process is completed in less than a day. The learning rate is set to 1e-3, and the dropout rate (0.2) is applied to prevent overfitting. The performance of the model is evaluated using appropriate evaluation metrics such as precision (*P*), recall (*R*), and *F*1-score (*F*). This experimental setup aims to leverage the power of the architecture and the CRF decoder to achieve accurate and robust NER across various biomedical datasets.

*Precision (P)*:


$$P=\frac{TP}{TP+FP}$$

*Recall (R)*:


$$R=\frac{TP}{TP+FN}$$

*F1-Score (F1)*:


$$F1=\frac{2\cdot P\cdot R}{P+R}$$

Where:


$$\begin{array}{c} TP-\text{True}\;\text{Positives}\;(\text{correctly}\;\text{predicted}\;\text{positive}\;\text{instances})\\ FP-\text{False}\;\text{Positives}\;(\text{negative}\;\text{instances}\;\text{incorrectly}\;\text{predicted})\\ FN-\text{False}\;\text{Negatives}\;(\text{positive}\;\text{instances}\;\text{incorrectly}\;\text{predicted}) \end{array}$$

### 3.3 Overview of results

The GRU-SCANET architecture outperformed the other approaches of the evaluated datasets (Table 1). With a model size of 16 million parameters, GRU-SCANET achieved metrics ranging from 83.52% to 98.64% (Table 3), consistently surpassing state-of-the-art models in various NER tasks. Notably, our architecture outperformed BioBERT in all eight evaluations and surpassed Bern2 in five out of five evaluations (Tables 3–5). Our analysis shows a robust balance between precision, recall, and F1-score, reflecting the model’s ability to accurately label entities in biomedical texts. For example, GRU-SCANET achieved an F1-score of 91.64% on the NCBI Disease dataset and 94.37% on the BC5CDR-chem dataset. These results indicate that GRU-SCANET effectively handles diverse biomedical NER tasks, ensuring high accuracy and minimal error rates. On the JNLPBA dataset, GRU-SCANET achieves a slightly lower precision (83.52%) than CRF (83.76%), which itself outperforms the other models evaluated in (Song *et al.* 2021; GRAM-CNN, Layered-BiLSTM-CRF, and MTM-CW). In terms of F1-score, GRU-SCANET surpasses all five models presented in (Song *et al.* 2021).

**Table 1.** Statistics of the biomedical NER datasets.^a^

Dataset	Entity Type	N-o-A
NCBI Disease (Doğan *et al.* 2014)	Disease	6881
BC5CDR (Li *et al.* 2016)	Disease	12 694
BC5CDR (Li *et al.* 2016)	Drug/Chem.	15 411
BC4CHEMD (Krallinger *et al.* 2015)	Drug/Chem.	79 842
BC2GM (Smith *et al.* 2008)	Drug/Chem.	20 703
JNLPBA (Collier *et al.* 2004)	Gene/Protein	35 460
LINNAEUS (Gerner *et al.* 2010)	Gene/Protein	4077
Species-800 (Pafilis *et al.* 2013)	Species	3708

a The provided information includes the number of annotations from Habibi *et al.* (2017) and Zhu *et al.* (2018). N-o-A: number of annotations.

**Table 2.** Performance metrics (F1: micro) achieved on benchmark datasets after progressive merging.^a^

Datasets	Precision	Recall	F1-score	Model’s size
D1	90.21	90.21	90.21	5659818 (6M)
D2	92.31	92.31	92.31	12133320 (12M)
D3	92.18	92.18	92.18	12566382 (13M)
D4	92.88	92.88	92.88	12566940 (13M)
D5	91.86	91.86	91.86	13039314 (13M)
D6	92.10	92.10	92.10	14189328 (14M)
D7	92.68	92.68	92.68	14458198 (14M)
D8	92.67	92.67	92.67	15079716 (15M)
D8 (no MHA)	57.90	57.90	57.90	10422436 (10M)

a Summary table of architecture performance after a progressive increase in data size. We also evaluated GRU-SCANET without the MHA layer that refers to D8 (no MHA). The highest performing scores are highlighted in bold, while the second-best scores are underlined.

**Table 3.** Results obtained from the evaluation of the biomedical named entity recognition system.^a^

				BERT	BioBert v1.0			BioBERT v1.1	GRU-SCANET
Type	Datasets	Metrics	SOTA	(Wiki + Books)	(+ PubMed)	(+ PMC)	(+ PubMed + PMC)	(+ PubMed)	
Disease	NCBI disease	P	88.30	84.12	86.76	86.16	89.04	88.22	**91.64**
		R	89.00	87.19	88.02	89.48	89.69	91.25	**91.64**
		F	88.60	85.63	87.38	87.79	89.36	89.71	**91.64**
	BC5CDR	P	89.61	81.97	85.80	84.67	85.86	86.47	**94.25**
		R	83.09	82.48	86.60	85.87	87.27	87.84	**94.25**
		F	86.23	82.41	86.20	85.27	86.56	87.15	**94.25**
Drug/Chem	BC5CDR	P	94.26	90.94	92.52	92.46	93.27	93.68	**94.37**
		R	92.38	91.38	92.76	92.63	93.61	93.26	**94.37**
		F	93.31	91.16	92.64	92.54	93.44	93.47	**94.37**
	BC4CHEMD	P	92.29	91.19	91.77	91.65	92.23	92.80	**92.83**
		R	90.01	88.92	90.77	90.30	90.61	91.92	**92.83**
		F	91.14	90.04	91.26	90.97	91.41	92.36	**92.83**
Gene/Protein	BC2GM	P	81.81	81.17	81.72	82.86	85.16	84.32	**89.47**
		R	81.57	82.42	83.38	84.21	83.65	85.12	**89.47**
		F	81.69	81.79	82.54	83.53	84.40	84.72	**89.47**
	JNLPBA	P	74.43	69.57	71.11	71.17	72.68	72.24	**83.52**
		R	83.22	81.20	83.11	82.76	83.21	**83.56**	83.52
		F	78.58	74.94	76.65	76.53	77.59	77.49	**83.52**
Species	LINNAEUS	P	92.80	91.17	91.83	91.62	93.84	90.77	**98.64**
		R	94.29	84.30	84.72	85.48	86.11	85.83	**98.64**
		F	93.54	87.60	88.13	88.45	89.81	88.24	**98.64**
	SPECIES-800	P	74.34	69.35	70.60	71.54	72.84	72.80	**95.72**
		R	75.96	74.05	75.75	74.71	77.97	75.36	**95.72**
		F	74.98	71.63	73.08	73.09	75.31	74.06	**95.72**

a Following the convention proposed by Lee *et al.* (2019) with their results, we adopt a similar approach to present our summary of results. For each dataset, we provide the Precision (P), Recall (R), and F1-score (F) scores but we use the average micro-metric to evaluate GRU-SCANET. The highest performing scores are highlighted in bold, while the second-best scores are underlined.

**Table 4.** Evaluations of the biomedical named entity recognition system (F1: micro).^a^

	BERT		RoBERTa	BioBERT	SciBERT		ClinicalBERT	BlueBERT	PubMedBERT	GRU-SCANET
Datasets	Uncased	Cased	Cased	Cased	Uncased	Cased	Cased	Cased	Uncased	
BC5-Chem	89.25	89.99	89.43	92.85	92.49	92.51	90.80	91.19	93.33	**94.37**
BC5-Disease	81.44	79.92	80.65	84.70	84.54	84.70	83.04	83.69	85.62	**94.25**
NCBI Disease	85.67	85.87	86.62	89.13	88.10	88.25	86.32	88.04	87.82	**91.64**
BC2GM	80.90	81.23	80.90	83.82	83.36	83.36	81.71	81.87	84.52	**89.47**
JNLPBA	77.69	77.51	77.86	78.55	78.68	78.51	78.07	77.71	79.10	**83.52**

a Comparison of GRU-SCANET’s results with those obtained by Gu *et al.* (2020) based on F1-score. The highest performing scores are highlighted in bold, while the second-best scores are underlined.

**Table 5.** Performance metrics (F1: micro) achieved on benchmark datasets for biomedical named entity recognition.^a^

Datasets	Type	PTC	Hunflair	Bern	Bern2	GSC
BC2GM	Gene/protein	78.8	77.9	83.4	83.7	**89.47**
NCBI Disease	Disease	81.5	85.4	88.3	88.6	**91.64**
BC4CHEMD	Drug/chemical	86.7	88.9	91.2	92.8	**92.83**
Linnaeus	Species	85.6	93.2	88.0	92.7	**98.64**
JNLPBA	DNA	N/A	N/A	N/A	77.8	**83.52**

a We incorporate our results into the existing version developed by Sung *et al.* (2022) for comparison and further analysis. GSC: GRU-SCANET. The highest performing scores are highlighted in bold, while the second-best scores are underlined.

A key observation is the equal number of false positives and false negatives across various test instances, leading to similar metrics for precision and recall. This balance highlights the model’s consistency and reliability in tagging entities. For example, if the model is expected to correctly tag 10 entities but makes one incorrect prediction, there will be 1 false positive (FP). Similarly, the number of false negatives (FN) will also be 1, indicating an incorrectly tagged position. When FN equals FP, precision (P) and recall (R) are equal. We calculate the average precision and recall over the entire test dataset, which leads to consistent metrics across different test instances (Lee *et al.* 2019).

Furthermore, the ablation study, which involved removing the Multi-Head Attention (MHA) layer, confirmed its crucial role in the model’s performance. The significant drop in F1-score to 57.90% without the MHA layer underscores its importance in capturing complex relationships between tokens. Overall, GRU-SCANET’s stable and high performance across different datasets and experimental setups showcases its capability to address the challenges of biomedical NER. This architecture sets a new benchmark for NER tasks in the biomedical domain, combining efficiency with accuracy to deliver state-of-the-art results.

### 3.4 Scalability and performance

**Figure 4.**
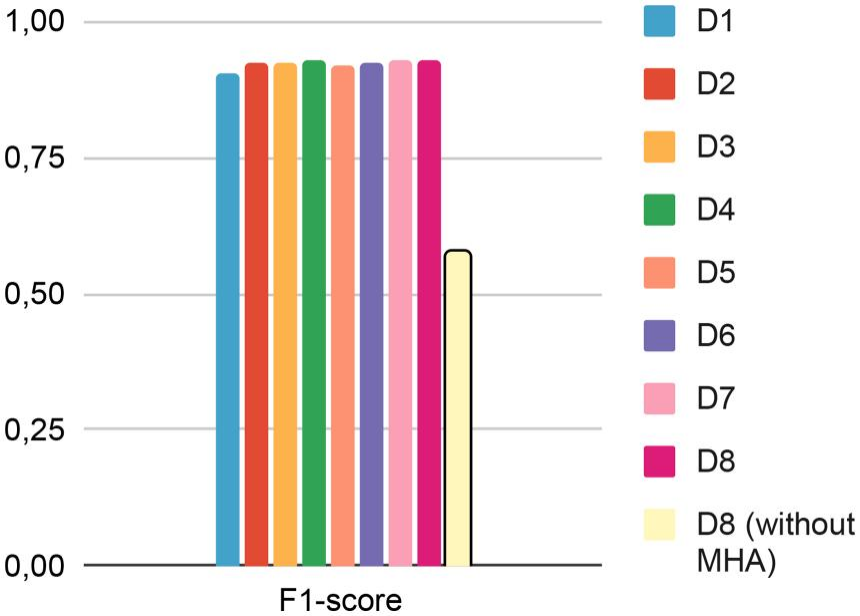
Performance comparison of models of different sizes obtained from the architecture.

**Figure 5.**
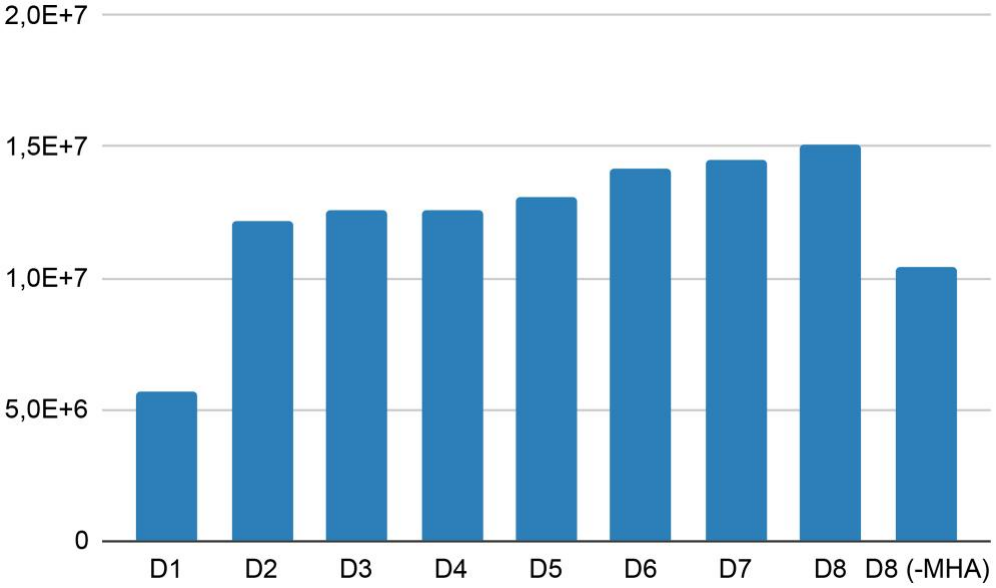
Parameter sizes for different models on datasets progressively merged.

**Figure 6.**
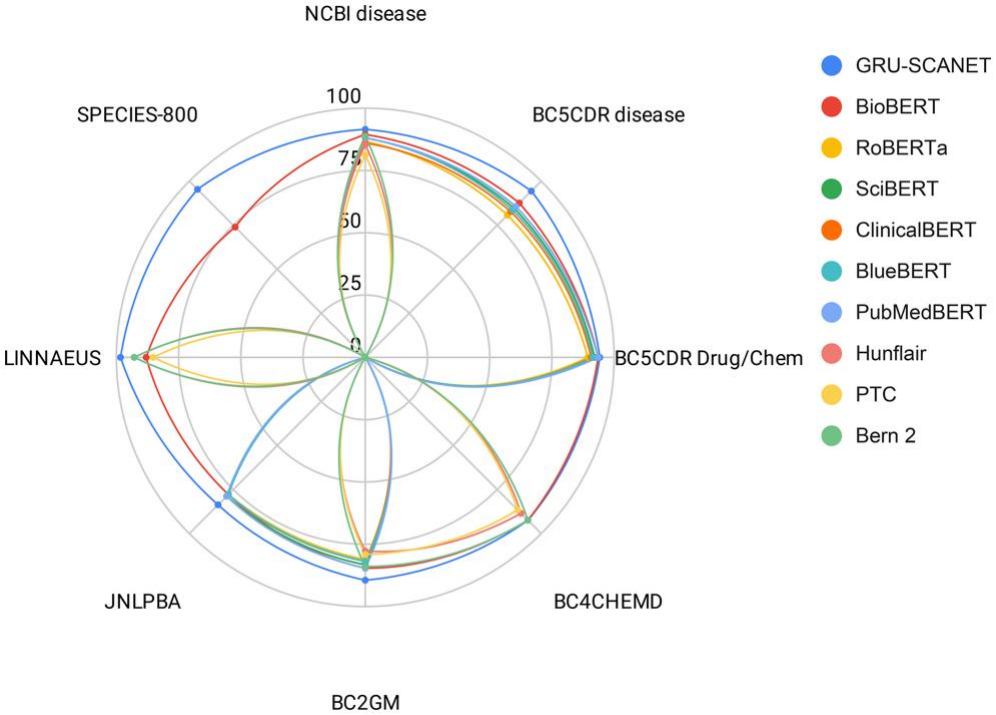
Summary of the performance of different models. The radar plot provides a simplified overview of F1-scores across all datasets.

Recall that we conducted evaluations on the following BioCreative datasets (8): BC2GM, BC4CHEMD, BC5CDR-chem, BC5CDR-disease, Corpora, JNLPBA, Linnaeus, NCBI Disease, and s800. Using these initial datasets, we created mergers of sets to construct datasets from D1 to D8. For instance, D3 comes from merging the first three datasets listed above. The results are recapitulated in Table 2. In Figs 4 and 5, after evaluating each model derived from these merged datasets with the architecture, we observe a stable and slightly increasing model size and stable performances from this architecture. Significant performance fluctuations across the different dataset combinations strongly indicate an imbalance in token distributions between the datasets. Nevertheless, the average value of all metrics is 92.11%. We also performed an ablation study of GRU-SCANET by removing the MHA layer and reevaluating the merged version of the entire dataset (D8). As shown in Table 2 (D8 without MHA), we observe a drastic drop in performance and reduced model size, confirming that the MHA layer is crucial and justifying our performance. Figure 6 highlights that GRU-SCANET globally outperforms other models in terms of F1-score on all datasets. This graph facilitates an intuitive and quick comparison of overall performance.

## 4 Discussion

Some studies have shown that it is highly beneficial for auto-regressive models to leverage external corpora tailored to specific domains to optimize token representations to increase the effectiveness in some tasks such as NER (Lee *et al.* 2019). This has been demonstrated by all models, whether they are of biological origin or not, that have adopted the BERT architecture [RoBERTa, BioBERT (cased, v1.0, v1.1), SciBERT, ClinicalBERT, BlueBERT, PubMedBERT, and Bern2]. However, in specialized, less-explored domains with a limited training corpus, stabilizing models that aim to optimize token vector representations across multiple contexts can be challenging due to the constraints of the dataset (Mikolov *et al.* 2013). Our approach GRU-SCANET can maintain high performance without extensive fine-tuning on domain-specific data. This is achieved using BiGRU for contextual learning, multi-head attention for capturing token relationships, and a CRF decoder for precise entity labeling. The ablation study further highlights the crucial role of the multi-head attention mechanism in achieving high accuracy, as evidenced by the significant drop in performance to 57.90% when this component is removed. The scalability of GRU-SCANET is another notable advantage. Our evaluations across progressively larger datasets demonstrate that the model’s performance remains stable and even improves slightly with increased data size. This indicates that GRU-SCANET can effectively handle the growing volume of biomedical literature, making it a robust tool for real-world applications. New approaches have emerged recently to highlight the ability of NER based on LLM prompt contexts with zero-shot or few-shot examples (Košprdić *et al.* 2023, Bian *et al.* 2023), which have so far provided almost no better results despite their varied performance.

## 5 Conclusion

In conclusion, this article introduces an architecture for biological NER that combines positional encoding, BiGRU, an attention-based encoder, and a CRF decoder. The experimental results validate the effectiveness of this approach in accurately identifying biological entities. Compared to existing models such as Bern, BERT, RoBERTa, BioBERT (cased, v1.0, v1.1), SciBERT, ClinicalBERT, BlueBERT, PubMedBERT, and Bern2, GRU-SCANET offers enhanced accuracy and efficiency in extracting named entities from biomedical texts. Evaluation results on benchmark datasets demonstrate the effectiveness of GRU-SCANET in recognizing various types of entities, including genes/proteins, diseases, drugs/chemicals, and species. Local installation options make GRU-SCANET easily accessible for integration into other systems. GRU-SCANET provides researchers and practitioners a reliable tool for improving biomedical text-mining tasks. Overall, GRU-SCANET holds great potential in advancing research and applications in biomedicine. Future perspectives for this approach include exploring larger and more diverse biomedical datasets, as well as incorporating domain-specific knowledge to improve performance in capturing specific biomedical entity types and relationships.

## Data Availability

Software, application demo, and source code are available at: https://github.com/ANR-DIG-AI/GRU-SCANET.

## References

[vbaf096-B1] Abdelgwad MM , Soliman THA, Taloba AI et al Arabic aspect based sentiment analysis using bidirectional GRU based models. J King Saud Univ, Comput Inf Sci 2022;34:6652–62.

[vbaf096-B2] Agarap AF. Deep learning using rectified linear units (ReLU). arXiv, arXiv:1803.08375, 2018, preprint: not peer reviewed.

[vbaf096-B3] Aguiar A , Silveira R, Furtado V et al Using topic modeling in classification of Brazilian lawsuits. In: *International Conference on Computational Processing of the Portuguese Language*. Springer, 2022, 233–42.

[vbaf096-B4] Al-Smadi M , Talafha B, Al-Ayyoub M et al Using long short-term memory deep neural networks for aspect-based sentiment analysis of Arabic reviews. Int J Mach Learn Cyber 2019;10:2163–75.

[vbaf096-B5] Bian J , Zheng J, Zhang Y et al Inspire the large language model by external knowledge on biomedical named entity recognition. arXiv, arXiv:2309.12278, 2023, preprint: not peer reviewed.

[vbaf096-B6] Bostrom K , Durrett G. Byte pair encoding is suboptimal for language model pretraining. In: *Findings of the Association for Computational Linguistics: EMNLP 2020*. Association for Computational Linguistics, 2020, 4617–24.

[vbaf096-B7] Catelli R , Gargiulo F, Casola V et al Crosslingual named entity recognition for clinical de-identification applied to a Covid-19 Italian data set. Appl Soft Comput 2020;97:106779. 33052197 10.1016/j.asoc.2020.106779 PMC7544600

[vbaf096-B8] Collie N , Ohta T, Tsuruoka Y et al Introduction to the bio-entity recognition task at JNLPBA. In: *Proceedings of the International Joint Workshop on Natural Language Processing in Biomedicine and its Applications (NLPBA/BioNLP)*. Geneva, Switzerland: COLING, 2004, 73–8. https://aclanthology.org/W04-1213.

[vbaf096-B9] Dai Z , Yang Z, Yang Y et al Transformer-XL: attentive language models beyond a fixed-length context. arXiv, arXiv:1901.02860, 2019, preprint: not peer reviewed.

[vbaf096-B10] Doğan RI , Leaman R, Lu Z. NCBI disease corpus: a resource for disease name recognition and concept normalization. J Biomed Inform 2014;47:1–10. 24393765 10.1016/j.jbi.2013.12.006 PMC3951655

[vbaf096-B11] Ehrmann M , Hamdi A, Pontes EL et al Named entity recognition and classification in historical documents: a survey. ACM Comput Surv 2023;56:1–47.

[vbaf096-B12] Forney G. The Viterbi algorithm. Proc IEEE 1973;61:268–78.

[vbaf096-B13] Gerner M , Nenadic G, Bergman CM. LINNAEUS: a species name identification system for biomedical literature. BMC Bioinformatics 2010;11:85. 20149233 10.1186/1471-2105-11-85 PMC2836304

[vbaf096-B14] Giorgi JM , Bader GD. Transfer learning for biomedical named entity recognition with neural networks. Bioinformatics 2018;34:4087–94. 10.1093/bioinformatics/bty449. 29868832 PMC6247938

[vbaf096-B15] Gu Y , Tinn R, Cheng H et al Domain-specific language model pretraining for biomedical natural language processing. 2020. CoRR, abs/2007.15779. https://arxiv.org/abs/2007.15779.

[vbaf096-B16] Günther M , Sikorski P, Thiele M et al Facete: exploiting web tables for domain-specific word embedding evaluation. In: *Proceedings of the Workshop on Testing Database Systems*, 2020, 1–6.

[vbaf096-B17] Habibi M , Weber L, Neves M et al Deep learning with word embeddings improves biomedical named entity recognition. Bioinformatics 2017;33:i37–48. 10.1093/bioinformatics/btx228. 28881963 PMC5870729

[vbaf096-B18] Joshi C , Attar VZ, Kalamkar SP. An unsupervised topic modeling approach for adverse drug reaction extraction and identification from natural language text[M]. In: *Advances in Data and Information Sciences*. Springer, 2022, 505–14.

[vbaf096-B19] Kiela D , Hill F, Clark S. Specializing word embeddings for similarity or relatedness. In: *Proceedings of the 2015 Conference on Empirical Methods in Natural Language Processing*, Lisbon, Portugal. Association for Computational Linguistics, 2015, 2044–8.

[vbaf096-B20] Košprdić M , Prodanović N, Ljajić A et al From zero to hero: harnessing transformers for biomedical named entity recognition in zero-and few-shot contexts. 2023. Available at SSRN 4463335. 10.1016/j.artmed.2024.102970 39197375

[vbaf096-B21] Krallinger M , Rabal O, Leitner F, et al The CHEMDNER corpus of chemicals and drugs and its annotation principles. J Cheminform 2015;7:S2. 25810773 10.1186/1758-2946-7-S1-S2 PMC4331692

[vbaf096-B22] Kratzert F , Gauch M, Nearing G et al Niederschlags-Abfluss-Modellierung mit long Short-Term memory (LSTM). Österr Wasser-Und Abfallw 2021;73:270–80.

[vbaf096-B23] Lafferty JD , McCallum A, Pereira FCN. Conditional random fields: probabilistic models for segmenting and labeling sequence data. In: *ICML ’01: Proceedings of the Eighteenth International Conference on Machine Learning*. San Francisco, CA, USA: Morgan Kaufmann Publishers Inc., 2001, 282–9.

[vbaf096-B24] Lai VD , Ngo NT, Veyseh APB et al ChatGPT beyond English: towards a comprehensive evaluation of large language models in multilingual learning. arXiv, arXiv:2304.05613, 2023, preprint: not peer reviewed.

[vbaf096-B25] Lee J , Yoon W, Kim S et al BioBERT: a pre-trained biomedical language representation model for biomedical text mining. Bioinformatics 2019;36:1234–40. 10.1093/bioinformatics/btz682. PMC7703786 31501885

[vbaf096-B26] Li J , Sun Y, Johnson RJ et al BioCreative V CDR task corpus: a resource for chemical disease relation extraction. Database (Oxford) 2016;2016:baw068. 27161011 10.1093/database/baw068 PMC4860626

[vbaf096-B27] Li J , Wei Q, Ghiasvand O et al A comparative study of pre-trained language models for named entity recognition in clinical trial eligibility criteria from multiple corpora. BMC Med Inform Decis Mak 2022;22:235–10. 36068551 10.1186/s12911-022-01967-7 PMC9450226

[vbaf096-B28] Li T , Zhang G, Do QD et al Long-context LLMS struggle with long in-context learning. arXiv, arXiv:2404.02060, 2024, preprint: not peer reviewed.

[vbaf096-B29] Liu AT , Xiao W, Zhu H et al QaNER: prompting question answering models for few-shot named entity recognition. CoRR, abs/2203.01543. 10.48550/arXiv.2203.01543, 2022.

[vbaf096-B30] Lo PS , Wu JL, Deng ST et al CNERVis: a visual diagnosis tool for Chinese named entity recognition. J Vis 2022;25:653–69.

[vbaf096-B31] Mikolov T , Sutskever I, Chen K et al Distributed representations of words and phrases and their compositionality. In: *Neural and Information Processing System (NIPS)*. 2013. https://papers.nips.cc/paper/5021-distributed-representations-of-words-and-phrases-and\\-their-compositionality.pdf.

[vbaf096-B32] Pafilis E , Frankild SP, Fanini L et al The SPECIES and ORGANISMS resources for fast and accurate identification of taxonomic names in text. PLoS One 2013;8:e65390. 23823062 10.1371/journal.pone.0065390 PMC3688812

[vbaf096-B33] Sapoval N , Aghazadeh A, Nute MG et al Current progress and open challenges for applying deep learning across the biosciences. Nat Commun 2022;13:1728. 35365602 10.1038/s41467-022-29268-7 PMC8976012

[vbaf096-B34] Shen Y , Yun H, Lipton ZC et al Deep active learning for named entity recognition. arXiv, arXiv:1707.05928, 2017, preprint: not peer reviewed.

[vbaf096-B35] Shrimal A , Jain A, Mehta K et al NER-MQMRC: formulating named entity recognition as multi question machine reading comprehension. In: *Proceedings of the 2022 Conference of the North American Chapter of the Association for Computational Linguistics: Human Language Technologies: Industry Track*. Hybrid: Seattle, Washington + Online: Association for Computational Linguistics, 2022, 230–8. https://aclanthology.org/2022.naacl-industry.26.

[vbaf096-B36] Smith L , Tanabe LK, Ando RJN et al Overview of BioCreative II gene mention recognition. Genome Biol 2008;9:S2. 10.1186/gb-2008-9-s2-s2 PMC2559986 18834493

[vbaf096-B37] Song B , Li F, Liu Y et al Deep learning methods for biomedical named entity recognition: a survey and qualitative comparison[J/OL.] Brief Bioinform 2021;22:bbab282. 10.1093/bib/bbab282. 34308472

[vbaf096-B38] Spositto OM , Bossero JC, Moreno EJ et al Lexical analysis using regular expressions for information retrieval from a legal corpus. In: *Argentine Congress of Computer Science*. Springer, 2022, 312–24.

[vbaf096-B39] Strobl M , Trabelsi A, Zaiane OR. Enhanced entity annotations for multilingual corpora. In: *Proceedings of the Thirteenth Language Resources and Evaluation Conference*, Marseille, France. European Language Resources Association, 2022, 3732–40.

[vbaf096-B40] Sung M , Jeong M, Choi Y et al Bern2: an advanced neural biomedical named entity recognition and normalization tool. arXiv, arXiv:2201.02080, 2022, preprint: not peer reviewed. 10.1093/bioinformatics/btac598 PMC9563680 36053172

[vbaf096-B41] Vaswani A , Shazeer N, Parmar N et al Attention is all you need. Adv Neural Inf Process Syst 2017;30.

[vbaf096-B42] Yoon W , So CH, Lee J et al CollaboNet: collaboration of deep neural networks for biomedical named entity recognition. BMC Bioinformatics 2019;20:249. 31138109 10.1186/s12859-019-2813-6 PMC6538547

[vbaf096-B43] Zhang L , Joho H, Fujita S et al Selectively expanding queries and documents for news background linking. In: *Proceedings of the 31st ACM International Conference on Information & Knowledge Management*. New York, NY, USA: ACM, 2022a.

[vbaf096-B44] Zhang X , Jiang Y, Wang X et al Domain-specific NER via retrieving correlated samples. In: Calzolari N, Huang C, Kim H, *et al. Proceedings of the 29th International Conference on Computational Linguistics, COLING 2022, Gyeongju, Republic of Korea*, October 12–17, 2022. International Committee on Computational Linguistics, 2022b, 2398–404. https://aclanthology.org/2022.coling-1.211.

[vbaf096-B45] Zheng Z , Lu X, Chen K et al Pretrained domain-specific language model for general information retrieval tasks in the AEC domain. CoRR, abs/2203.04729. 10.48550/arXiv.2203.04729, 2022.

[vbaf096-B46] Zhu H , Paschalidis IC, Tahmasebi AM. Clinical concept extraction with contextual word embedding. In: *NIPS Machine Learning for Health Workshop*. 2018. https://par.nsf.gov/biblio/10098080.

